# Hormonal Changes in Menopause and Orexin-A Action

**DOI:** 10.1155/2013/209812

**Published:** 2013-06-11

**Authors:** Giovanni Messina, Andrea Viggiano, Vincenzo De Luca, Antonietta Messina, Sergio Chieffi, Marcellino Monda

**Affiliations:** ^1^Department of Experimental Medicine, Section of Human Physiology and Clinical Dietetic Service, Second University of Naples, Via Costantinopoli 16, I-80138 Naples, Italy; ^2^Faculty of Medicine, University of Salerno, Salerno, Italy

## Abstract

Menopause is a period of significant physiological changes that may be associated with increased body weight and obesity-related diseases. Many researches were conducted to assess the contribution of factors such as estrogen depletion, REE decline, and aging to weight gain. An increase in orexin-A plasma levels, paralleling lower estrogen levels, was found during menopause. Orexins are hypothalamic neuropeptides recently discovered, involved in the regulation of feeding behaviour, sleep-wakefulness rhythm, and neuroendocrine homeostasis. Orexins might offer the missing link between postmenopausal hypoestrogenism and other manifestations of the menopausal syndrome, including appetite and weight changes and increase in cardiovascular risk.

Menopause is a period of significant physiological change that is largely related to estrogen depletion and subsequent cessation of ovarian function. During menopause period, women tend to gain weight and FM (fat mass) [1]. It is not clear whether the increase in adiposity is a consequence of the decline in endogenous estrogen. Several studies faced the question by using postmenopausal hormone-replacement therapy (HRT). If the increase in adiposity is a consequence of the decline in endogenous estrogen that occurs at this time, HRT should prevent or reduce body fat gain. However, existing clinical data addressing this issue are discordant. Anderson et al. [2] showed that short-term (2 months) use of HRT did not alter body mass index (BMI), FM, or fat-free mass (FFM) in postmenopausal women [3]. With long-term use (1 year), Reubinoff et al. [4] found a similar increase in body weight and FM among women taking HRT and those who declined its use. They did, however, observe that there was a significant shift from gynoid to android fat distribution only in women not taking HRT [5]. A decrease in body weight was found by Espeland et al. [6] over a 3-year period in women taking HRT compared to women not taking HRT. Conversely, other data suggested that oral estrogen might cause an increase in body fat, possibly by limiting lipid oxidation [3, 7]. Thus, whether and how hormone therapy affects body composition in postmenopausal women is still unclear. Ovarian hormones may influence body composition through several potential mechanisms. It has been suggested that estradiol inhibits the action of adipose tissue lipoprotein lipase, the enzyme that hydrolyzes circulating triglycerides, allowing for the uptake of fatty acids into adipocytes [5]. Data from rodent models indicate that estrogen acts as an anorectic, decreasing voluntary food intake [8]. 

Further, weight gain in postmenopausal women may depend on an accelerated resting energy expenditure (REE) decline [9, 10]. In this regard, it was found that REE declines by approximately 420 kcal/day in postmenopausal women compared with premenopausal women [11]. 

REE accounts for 60–75% of total daily energy expenditure. Various factors contribute to the interindividual variability in REE such as FFM [12], sympathetic nervous system (SNS) activity [13, 14], and endocrine status (e.g., thyroid hormone [15]). REE decreases with age [16, 17]. The age-related decline in REE could be due not only to the loss of FFM and an alteration in its metabolically active components, but also to reduction in physical activity. It is well known that the reduction in physical activity leads to a reduction in REE and a decrease in FFM. 

The decline in REE observed in postmenopausal women may depend on aging. However, REE seems to decrease more during the menopause transition than could be attributed to the aging process [18]. Estrogen depletion probably contributes to accelerated REE decline. Experimental evidence showed that estrogen increases physical activity-related energy expenditure [19, 20]. During the menopause transition, the decrease in REE accelerates the gains in FM which, in turn, may contribute to increasing the incidence of obesity-related diseases such as a worsening of cardiovascular risk profile [1, 21] and type II diabetes [18]. Also, estrogen depletion by itself seems to increase cardiovascular risk [22–24]. Staessen et al. [22] observed that the incidence of hypertension was significantly higher in hypoestrogenic postmenopausal women when compared with women receiving HRT, after adjustment for age, race, and weight. Comparable findings were reported by Vongpatanasin et al. [23] and Weitz et al. [24] in their studies, concluding that HRT lowered diastolic blood pressure in postmenopausal women. Regarding the metabolic variables evaluated, it was found that postmenopausal women not receiving HRT had significantly higher plasma cholesterol and TG levels than reproductive-age women, but, more importantly, the levels were also higher than in those receiving HRT [25]. It has been observed that in postmenopausal women, plasma orexin-A levels were significantly higher, paralleling the significantly lower estrogen levels [25]. This aspect was found by El-Sedeek et al. [25] who assessed plasma orexin-A levels and estradiol in a group of postmenopausal women not receiving HRT and compared the values with a group on HRT and a group of reproductive-age women. The results showed that postmenopausal women not receiving HRT had the highest levels of plasma orexin-A. Conversely, postmenopausal women on HRT had orexin-A levels that were comparable with the control group. However, it should be noted that the determination of plasma orexin-A levels presents evident difficulties being such levels extremely low.

Further, the authors found that plasma orexin-A levels were directly correlated with some cardiovascular risk factors, namely, blood glucose, and lipid profile, arterial blood pressure, and body mass index. It has been suggested that plasma orexin-A levels parallel plasma estradiol levels as orexin is a central metabolic fuel detector, and physiological mechanisms that control energy balance are reciprocally linked to those that control reproduction. Further, orexin-A seems to play an important role in the control of the hypothalamic pituitary-gonadal axis [26], and gonadotropin-releasing hormone (GnRH) neurons in the hypothalamus have been found to be receptive to orexin modulation [27]. 

Experimental evidence suggests a mutual regulation of orexin and sexual steroids secretions. It has been reported that intracerebroventricular administration of orexin-A stimulates LH secretion in castrated female rats primed with estradiol and progesterone and inhibits LH secretion in unprimed rats. This dual effect may be due to a steroid regulation of the orexin receptors in selected areas, as the hypothalamus and the adenohypophysis [28, 29].

Orexins A and B (also known as hypocretins 1 and 2) are hypothalamic neuropeptides recently discovered, involved in the regulation of feeding behaviour, sleep-wakefulness rhythm, and neuroendocrine homeostasis [30–32]. Orexins promote both waking and feeding [33]. In addition to this central role, orexins probably have peripheral effects. This was suggested by the detection of substantial levels of orexins in plasma [34], as well as the demonstration of orexin receptors in several peripheral tissues, including the gastrointestinal tract (GIT), endocrine pancreas, adrenal glands, and adipose tissue [35, 36]. Snow et al. [37] have demonstrated that plasma orexin levels are one-fifth to one-eighth of orexin cerebrospinal fluid (CSF) values. It has been demonstrated that orexin-A influences several physiological variables. An intracerebroventricular (ICV) injection of orexin-A induces an increase in heart rate [38], blood pressure [39], and metabolic rate [40], thus, indicating that this neuropeptide plays a role in the control of vegetative functions. The expression pattern of mRNA encoding two orexin receptors (OX1R and OX2R) in the rat's brain has been demonstrated by Trivedi et al. [41]. Within the hypothalamus, expression for the OX1R mRNA was largely restricted in the ventromedial (VMH) and dorsomedial hypothalamic nuclei, while high levels of OX2R mRNA were contained in the paraventricular nucleus, VMH, and arcuate nucleus, as well as in mammillary nuclei [42]. Lu et al. [43] have showed that levels of OX1R mRNA were significantly increased in the VMH of rats after 20 h of fasting. An initial decrease (14 h) and a subsequent increase (20 h) in OX1R mRNA levels after fasting were observed in the dorsomedial hypothalamic nucleus. Levels of OX2R mRNA were augmented in the arcuate nucleus, but remained unchanged in the dorsomedial hypothalamic nucleus and paraventricular hypothalamic nucleus following fasting. The time-dependent and region-specific regulatory patterns of OX1R and OX2R suggest that they may participate in distinct neural circuits under the condition of food deprivation. These evidences suggest that the two types of orexin receptors are involved in different responses. In addition, the presence of orexin receptors in other cerebral areas suggests that additional functions are played by orexin-A [31]. A role for the orexins in sleep regulation has been demonstrated [44]. Deficiency in orexin neurotransmission results in the sleep disorder narcolepsy in mice, dogs, and humans [45]. Orexin-A also influences body temperature. In fact, an ICV administration of orexin-A induces an increase in firing rate of the sympathetic nerves to interscapular brown adipose tissue (IBAT), accompanied with a rise in IBAT and colonic temperatures [46]. The simultaneous increase in heart rate and body temperature after an ICV injection of orexin-A indicates a generalized activation of the sympathetic nervous system. Few investigations have been performed on the role played by different cerebral areas involved in the induction of the abovementioned tachycardia and hyperthermia [47–49]. The VMH controls thermogenic responses through the autonomic nervous system [50]. Electrical or chemical stimulations of the VMH increase the temperature of IBAT that is the principal effector of nonshivering thermogenesis [38, 51]. Lesions of the VMH reduce the sympathetic activation and thermogenic changes induced by various stimuli [52]. The hyperthermia due to sympathetic and thermogenic ICV injection of prostaglandin E1 is reduced by ibotenate lesion of the VMH [46]. Postingestional thermogenesis is lowered by the VMH lesion [53, 54]. This evidence indicates that the VMH is involved in hyperthermic reactions induced by several stimulations [55–57]. Orexin-A may partially mediate pressor response by increasing basal sympathetic activity, causing catecholamine release, modulating the vasopressin system [58], and stimulating renal and adrenal orexin receptors [59]. These speculations are further supported by Shiraska et al.'s study [39], where experimental use of orexin-A has been shown to increase heart rate, renal sympathetic activity, catecholamine release, and mean arterial blood pressure. Orexins have been shown to adversely affect the plasma lipoprotein profile [60] and insulin glucose homeostasis and to stimulate insulin release from pancreatic cells in vivo and in vitro [61]. Orexin derangements in patients with narcolepsy were associated with an increased BMI [62] and a higher risk of type-II diabetes mellitus [63]. The influence of orexin-A on sympathetic nervous system activity, blood pressure regulation, and metabolic status may contribute to the postmenopausal upsurge in cardiovascular morbidity and mortality [25]. Conversely, estrogen playing a possible inhibitory effect on orexin might partially account for its cardioprotective effect [25]. A better and deeper understanding of orexins' effects could provide a new interpretation of the relationship between postmenopausal hypoestrogenism and menopausal syndrome, including appetite and weight changes, increased cardiovascular risk, vasomotor symptoms, and sleep disorders. 

Recently, Herring et al. [64] evaluated the utility of suvorexant, an orexin receptor antagonist, for treating patients with primary insomnia. Patients received suvorexant in one period and placebo in the other. Suvorexant showed significant dose-related improvements versus placebo on sleep efficiency. Thus, the authors [64] suggested that orexin receptor antagonists may represent a novel and useful approach to treat insomnia.

Further, the finding of a relationship between estrogen and orexins levels also suggests new research on the possible roles of orexins in many reproductive abnormalities and anomalies involving REE.
